# Automatic Stomatal Segmentation Based on Delaunay-Rayleigh Frequency Distance

**DOI:** 10.3390/plants9111613

**Published:** 2020-11-20

**Authors:** Miguel Carrasco, Patricio A. Toledo, Ramiro Velázquez, Odemir M. Bruno

**Affiliations:** 1 Facultad de Ingeniería y Ciencias, Universidad Adolfo Ibañez, Av. Diagonal Las Torres, 2700 Santiago, Chile; patricio.toledo@uai.cl; 2 Facultad de Ingeniería Josemaría Escrivá de Balaguer 101, Campus Aguascalientes, Universidad Panamericana, Aguascalientes 20290, Mexico; rvelazquez@ags.up.mx; 3 Scientific Computing Group, São Carlos Institute of Physics, University of São Paulo, P.O. Box 369, São Carlos, SP 13560-970, Brazil; bruno@ifsc.usp.br

**Keywords:** stomatal segmentation, image segmentation, Delaunay-Rayleigh frequency

## Abstract

The CO_2_ and water vapor exchange between leaf and atmosphere are relevant for plant physiology. This process is done through the stomata. These structures are fundamental in the study of plants since their properties are linked to the evolutionary process of the plant, as well as its environmental and phytohormonal conditions. Stomatal detection is a complex task due to the noise and morphology of the microscopic images. Although in recent years segmentation algorithms have been developed that automate this process, they all use techniques that explore chromatic characteristics. This research explores a unique feature in plants, which corresponds to the stomatal spatial distribution within the leaf structure. Unlike segmentation techniques based on deep learning tools, we emphasize the search for an optimal threshold level, so that a high percentage of stomata can be detected, independent of the size and shape of the stomata. This last feature has not been reported in the literature, except for those results of geometric structure formation in the salt formation and other biological formations.

## 1. Introduction

Stomata are the gates through which gas exchange through the leaf takes place, involving carbon dioxide intake and water vapor loss (reviewed in Reference [1,2,3]). The carbon dioxide intake drives plant growth and productivity [4], while the water vapor loss through a process known as transpiration regulates leaf temperature, nutrient uptake, and root-to-shoot signaling [5,6]. The remaining of the epidermis is covered by an impervious cuticle, with restricted gas exchange properties [7,8]. Gas-exchange capacity depends on stomatal density (i.e., stomata number per unit area), size, and patterning (spacing) [9,10,11]. Methods for automatic segmentation of stomatal density and size have been earlier presented [12,13], whereas patterning (spacing) is currently performed manually [14].

From a computational point of view, stomatal analysis has been explored mainly on pixel or object analysis. Although localization and processing tools exists, these are limited to search for stomatal position through techniques based on chromatic and morphological combinatorial operations [15,16,17], texture analysis [16], fractal analysis [18], segmentation using object-oriented method in multiple resolutions [19], and, more recently, by Deep Convolutional Neural Networks [12,13,20,21,22]. Today, no technique allows full spectrum analysis of different stomatal types since they have a great variation according to species, shape, position, and, in general, noise and technique present in the microscopy image [23,24,25,26]. At large, both the location and the analysis of the stomata require demanding work on the part of the biologists. This task is not simple, since there are great differences in the same species by the microscopy technique used [27]. This research proposes a new segmentation algorithm based on the spatial stomatal statistical distribution. We define a geometric model, revisited in recent years, implying a mechanism present in nature and allowing an ideal distribution for gas transfer [5,14,28].

The study and analysis of stomata is the first step towards phenotypic plasticity and the relationship with climate change, soil use, resource, drought, CO_2_ concentration, and light level [4,8,29,30,31,32,33]. Consequently, segmentation process automation and stomatal morphology analysis is a relevant task, especially in high volume analysis of images needed as input for other studies [20]. The latter might not be viable by hand because of analysis complexity. Although we use a specific microscopy database, both for the design and for the analysis of the computational algorithm, our findings have potential use for any kind of stomatal images since it uses as key information stomata’s geometric configuration between analyzed species. We generate a manual segmentation in different species of *monocot* and *dicot* families to test our hypothesis. The results section presents the main findings of our algorithm through a fully automatic system for the *Jatoba Hymenaea Courbari* species. Subsequently, we manually analyze the algorithm proposed in other species in order to understand the existence of an underlying pattern between them. The Materials and Methods’ section presents a description of the steps of the algorithm and, finally, the general conclusions of the method.

## 2. Results

Delaunay-Rayleigh Threshold Binarization (DRTB) algorithm has been evaluated over a set of 31 optic microscopy images from *Jatoba Hymenaea Courbaril* tree localized in Caribbean, Central and South American zones. The images were taken with an Olympus E-330 camera with optic microscope with 3136 × 2352 resolution pixels, at the Biosciences Institute at University of São Paulo, Brazil (USP). The images’ set has 3087 regions hand-classified as stomata (see Supplementary Materials section) with its coordinates (*x*,*y*).

The abaxial surfaces of the epidermis were obtained from three or four leaves (one per individual) of each species. From each collected leaf, a sample of approximately 1 cm^2^ was removed from the leaf’s middle region, between the margin and midrib. Dissociation of the leaf epidermis was performed using a 1:1 solution of glacial acetic acid and hydrogen peroxide at 60 °C for 12 h, or the time required to completely decouple the epidermis [34].

**Figure 1:**
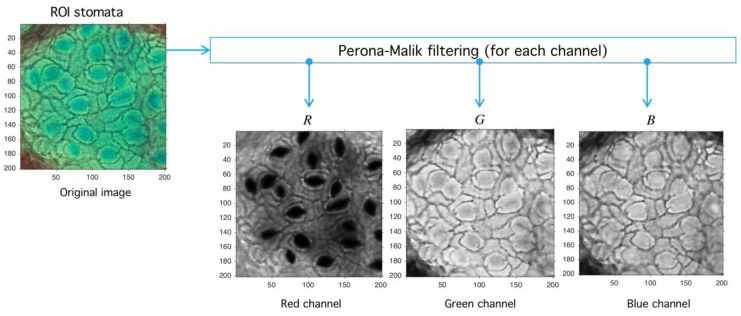
Illustration of precision and recall concepts. True positive (*TP*) occurs when the distance between manual-coordinate (yellow cross) is less than a threshold with respect to the coordinate of the segmented-region (ellipses). False negative (*FN*) occurs when the algorithm does not find the stoma, even when it is present in the image. Finally, when the algorithm classifies a region where there is no stoma, we consider this coordinate as False Positive (*FP*). The ratio *TP*/(*FP* + *TP*) is known as precision (positive predictive value (*PPV*)), and the ratio (*TP*/*TP* + *FN*) is called recall (true positive rate (*TPR*)).

The algorithm’s performance was evaluated by two statistical indicators: precision (also known as positive predictive value, *PPV*) and recall (or true positive rate, *TPR*). The procedure consists of manually identifying stoma’s center (*x*,*y*) coordinates. Then, the segmentation-algorithm identifies this position and proceeds with a comparison. If the distance between the manual coordinate is less than a threshold with respect to the coordinate of the segmented region, we consider that it has been classified correctly. This case is considered as True Positive (*TP*). In case the algorithm does not find the stoma, even when it is present in the image, we consider this case as False Negative (*FN*). Finally, when the algorithm classifies a region where there is no stoma, we consider this coordinate as False Positive (*FP*) (see Figure 1). Therefore, the evaluation metrics are:

(1) $$PPV\;=\;\frac{TP}{FP+TP},$$

(2) $$TPR\;=\;\frac{TP}{TP+FN}.$$

**Figure 2:**
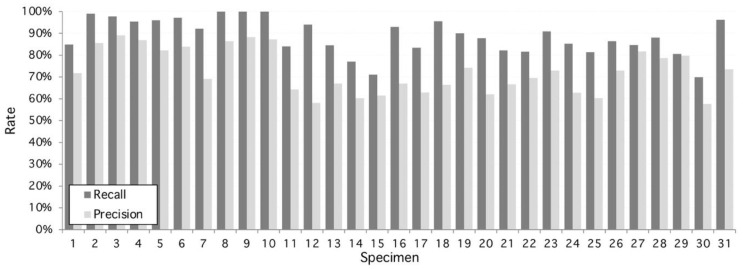
Performance of the proposed algorithm applied to 31 specimens of *Hymenaea Courbaril* (Jatoba Database). Overall recall and precision are 89.14% and 72.8%, respectively. Maximum recall performance is achieved at specimens #8, #9, and #10 with 100% and maximum precision performance is reached at 98% by specimen #3. Worst recall and precision performance is 70% and 58%, respectively, both at specimen #30.

**Figure 3:**
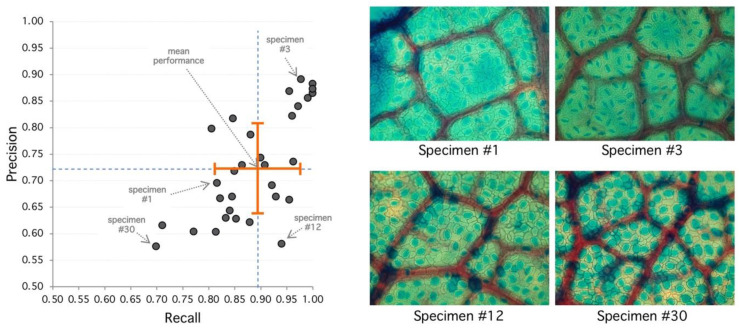
Analysis of selected specimens. (Left) Best performance is achieved at the upper-right area of the diagram with high recall and precision. Worst performance is achieved at the lower-left area. Orange cross indicates mean (recall/performance 83%/72%) and standard deviation (8%/10%). (Right) Specimen #1 represents an average performance (85%/72%) with clear regions but high diffusion. Specimen #3 has 98%/89% detection performance, and stomata shows sharp borders with clear regions leading to good detection metrics. Specimen #12 shows 94%/58% with high recall and lower precision, explained by diffuse borders and poor region definition. Specimen #30 has the poorest performance with 70%/58% with very diffuse borders and poor regions with high false positive rate.

**Figure 4:**
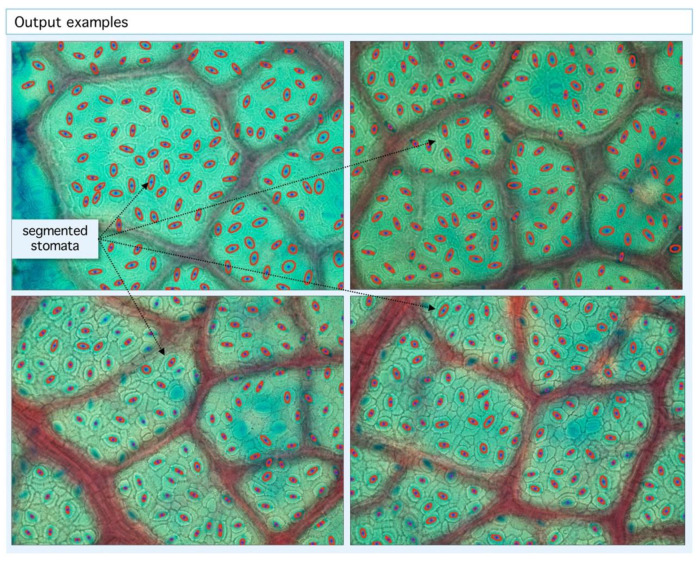
Example of segmentation output. Our algorithm is able to detect stomatal centroids (blue dots) and segmented areas (red ellipses). With the geometric information from centroid coordinates, statistics and tessellations are built.

From this set, the proposed algorithm is capable of 2752 detections (recall) of 89.14 ± 8%. On the other hand, the number of false positive regions (precision) is 72.8 ± 10%. As shown on Figure 2, the performance is variable with each sample, given structural conditions; however, a high classification rate is achieved as a result of multilevel threshold traveling design (see examples in Figure 3 and Figure 4). In contrast, lower results occur at lower image quality where the stomata are located because they are out of focus. The combined performance of both measures reflects this fact, and it is shown in Figure 3 (specimen).

## 3. Discussion

**Figure 5:**
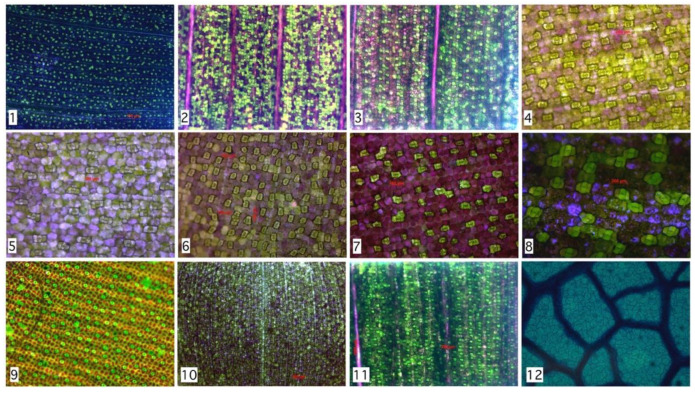
Hand segmentation to 2842 stomata within 12 species as described in Table 1. Despite morphological and chromatically differences, the spatial distributions are similar to the example shown in Figure 4 (*Hymenaea Courbaril*).

Previous results reveal a high precision rate in the detection of *Hymenaea Courbaril* stomata. However, is this distribution similar on other species? To answer this question, we analyzed seven species with different microscopy techniques. Experimental results show that not only *Hymenaea Courbaril* has a stable distribution, we applied a manual segmentation to 2842 stomata with 12 different configurations shown in Figure 5 and described in Table 1. Despite morphological and chromatic differences, the spatial distributions are similar, as it is shown in Figure 4. These results are consistent with the findings found by Croxdale [9], where the relationship between area and number is analyzed in the first instance. This result is relevant since it provides statistical evidence that would allow us to design an optimal algorithm for the relative distance between stomata, which is consistent with Peat & Fitter [35] developments. The only requirement is a preprocess with efficient acceptance/rejection range for stomatal segmentation. This last task could be performed by any segmentation algorithm, such as those mentioned above. It is known that environmental conditions during growth may also affect stomatal dispersion pattern among species [36,37]. Based on this background, stomatal spacing may be employed as a marker for studying plant adaptation to growth environment [38]. Next, we present a brief discussion of manual segmentation in different types of plants of the *Monocot* family, compared to the automatic evaluation of the *Dicot* family.

### 3.1. Manual Segmentation

The automatic algorithm evaluation gives TPR=89.14±8% and PPV=72.8±10%. Those results are relevant given image type. Our results are statistically valid, considering the total number of stomata studied. To assess if this relation is relevant for other species, we evaluate 12 different configurations on 7 different plant species. Despite interspecies variations, a relevant result is the Root-Mean-Square Deviation (RMSD) falling with segmented region number. In terms of distribution parameters, they are stable in the range 3<σ<8  as long as region number is lower than 100. As future work, it remains to design an optimal search algorithm based on a distribution parameter in the appropriate range, depending on the type of species, the analysis of second (autocovariance) and higher order moments of geometric characteristics, and the cross covariances with other relevant properties captured by microscopes, like color.

**Figure 6:**
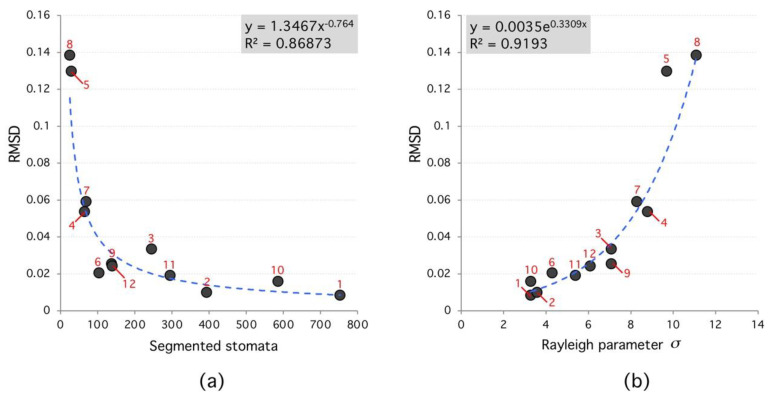
Root-Mean-Square Deviation (RMSD) versus segmented stomata number and Rayleigh parameter. (**a**), the RMSD decreases as a power law with number of segmented stomata. (**b**), RMSD increases exponentially with Rayleigh parameter. High RMSD species (red labels) tend to have lower number of segmented regions and higher Rayleigh parameter.

**Figure 7:**
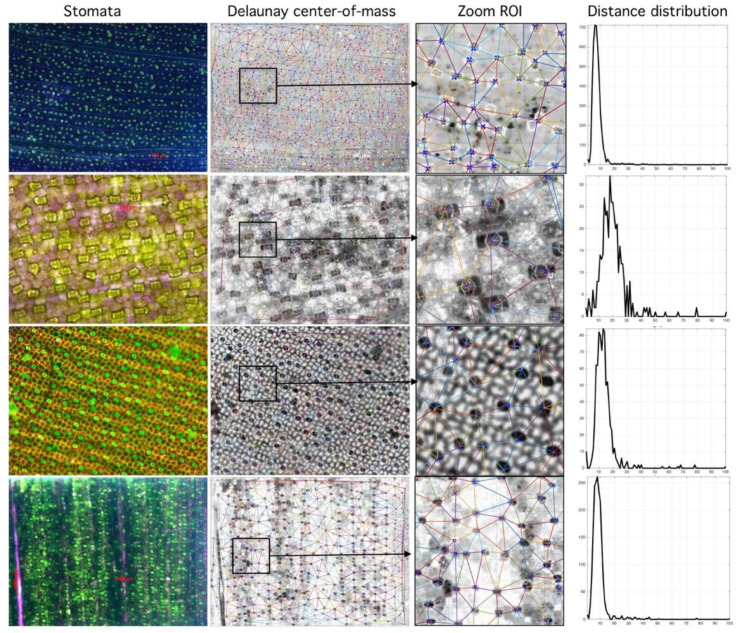
Relationship between segmented region and Rayleigh distribution histogram. First column (stomata) shows different species analyzed. Second column (Delaunay center-of-mass) shows mass centers and corresponding tessellations. Third column (Zoom Region-of-Interest, ROI) shows region of interest. Fourth column (Distance distribution) shows sensibility of histogram to spatial distribution (more at https://github.com/mlacarrasco/drtb/tree/main/stomatasDB/output).

According to the analyzed data, the segmented region number affects the algorithm performance. In general, the higher the segmented region number the lower RMSD between Rayleigh distribution and frequency histogram; which confirms the true nature of the spatial distribution of stomata (see Figure 6). Moreover, Rayleigh distribution ideal parameters (that minimize RMSD) have a narrow range between 3 < σ < 8; see Figure 7. For each species, this parameter is different; however, results tend to cluster as the segmented region number reaches 100.

### 3.2. Defocus Stomata in Automatic Mode

**Figure 8:**
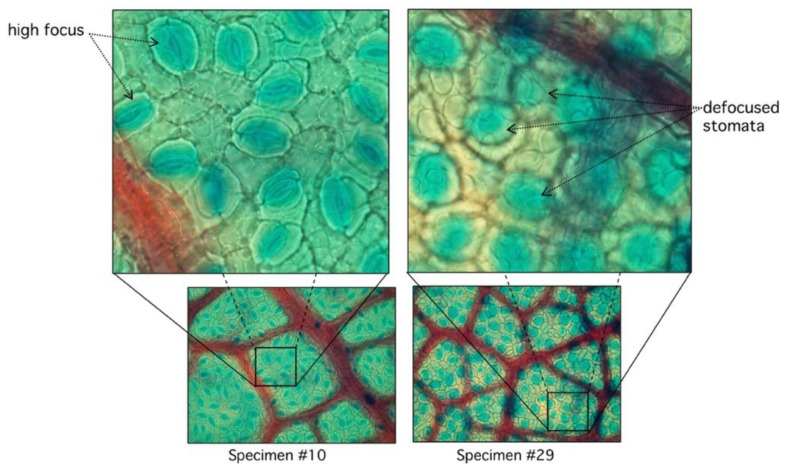
Comparison between high and low focused stomata. In specimen #10, stomata are clearly visible with sharp edges with very low RMSD (see Figure 7). Specimen #29 has diffused edges.

In the automatic segmentation mode, we observe relevant differences in the maximum and minimum performance of the proposed algorithm. When analyzing these differences in detail, we observe that they are due to the lack of focus of the stomata in some samples of the study (Figure 8).

### 3.3. Main Advantages and Disadvantages

As a first advantage, we highlight that our method is based on the stomatal geometric properties, easily verifiable, such as centroids spatial distribution; which does not depend on the shape and size of the stomata. Second, the image-preprocessing method is a well-established procedure in the field of imaging, and it is inspired by the property of diffusion, which is one of the fundamental mechanisms of stomatal physiology. Third, stomatal geometry is described by a Delaunay tessellation, which is a technic known to captures diffusive-processes, such as those occurring at the cellular level. Fourth, the use of the median as the main statistic decreases outliers-weight, which increases robustness. Regarding the disadvantages, our proposal might be classified as standard as it was not implemented through a deep-learning scheme (although it would be possible to do so). Despite our efforts to capture a large number of samples, we recognize that the number of species is limited, although high variability between samples can be observed. Regarding the distribution’s discrimination method, it is known that RMDS has some bias, which could be avoided with an Akaike scheme or similar. The preprocessing phase of our proposal is designed for *Hymenaea Courbaril*, as future work, and this process might be automated in such a way that it is possible to accept segmentation in other types of stomata.

## 4. Materials and Methods

**Figure 9:**
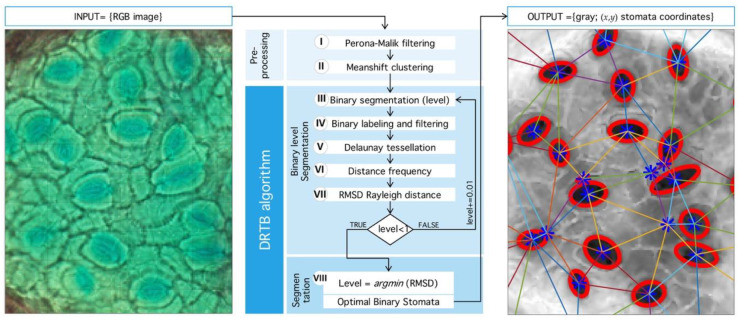
General process implemented in detection algorithm. (Left) Input RGB image from microscope. (Center) Sequence of processing: First, Preprocessing stage: Perona-Malik filtering followed by Meanshift clustering. Second, segmentation algorithm proposed (DRTB): binarization, labeling, tessellation, distance analysis, and segmentation stage plus optimal leveling. (Right) Final output image with segmented region and centroid based tessellation.

The natural way to deal with structural complexity found in stomata images is noise analysis. The latter is associated, by large, to simplification and information-reduction processes, like anisotropic diffusion, wavelet transform techniques, and nonlinear, statistical, or adaptive filters [39,40,41]. Even when image segmentation is possible, for example, by morphological processing, the use of geometric properties is desirable. Therefore, we propose a novel segmentation method that seeks an optimal binarization threshold through Rayleigh-distribution distance-minimization. This idea was inspired by Staff et al. [42], where they analyzed stomatal pore center coordinates, but, in this case, only areas of the inner triangles and their angles were reported. In Reference [43], a statistical mechanics explanation of this phenomenon is given through particle interaction, in this case, the distance between stomata, as a means of regulation and state of the tissue in general. An algorithm summary is shown in Figure 9.

### 4.1. Preprocessing

Standard noise-reduction and boundary-preservation techniques are used given the common noise properties present in most stomata images. We propose a two-step methodology based on Perona-Malik (PM) diffusion and Meanshift-Hadamard intensity-reduction.

**Step I.** Perona-Malik: The first step is PM iterative filter application [44]. This algorithm reduces noise level preserving and, at the same time, boundary structure through diffusion adaptation [45], meaning a lower diffusion constant near boundaries, and higher otherwise. Being an interactive filter, level simplification of image structures is possible, which is a leading characteristic in stomata search. Filter design criteria defined by Perona and Malik [46] are causality, immediate localization, and piecewise smoothing. The last three properties are relevant in our problem given that stomata have well defined structures hard to segment, mainly by their surrounding structures. As for causality, PM filters all regions classified as noise. Immediate localization allows sharp boundaries, despite scale changes, and the most useful property here is piecewise smoothing since it allows smaller structures to collapse into larger ones sharing a visual similarity criterion.

**Figure 10:**
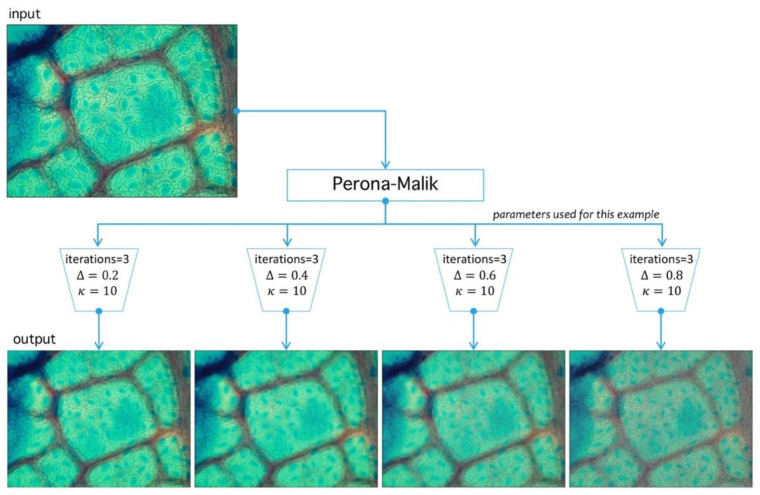
Image preprocessing with Perona-Malik (PM) filtering. PM has three main parameters: Δ, which represents the diffusion level; κ, which represents an advance-step (time in the original PDE framework); and the iteration number (advance-step times iteration number is the total time in the PDE framework). Four examples are shown to exemplify diffusion action over an image; higher Δ parameter means more diffuse image.

**Figure 11:**
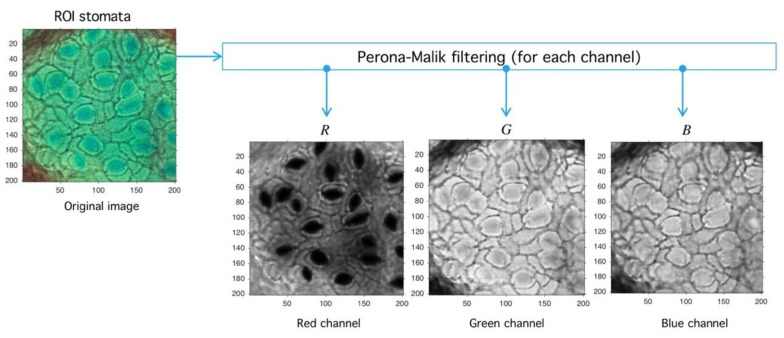
Image preprocessing with Perona-Malik (PM) filtering. After the PM usage (see Figure 10) the image is decomposed into red, green, and blue channels (RGB decomposition) with a standard routine (see shared code at GitHub). The red channel $P^{R}$ is used later in the next process.

Let P be an image defined over the space $M_{n,m}^{3}\left(\mathbb{N}\right)$ of arrays with n rows and m columns with pixels in the natural numbers. Depending on the color-space, {Red, Green, Blue} (RGB) or {Hue, Saturation, Value} (HSV)superscripts are used to refer the components. The PM filter $\partial_{t}$ defined from $M_{n,m}\left(\mathbb{N}\right)$ into itself is a solution of the heat-equation parameterized by t representing the number of times the filter is applied (time in the original partial differential equation context) and a diffusion parameter controlling the noise level (see Reference [46] for more details) (see Figure 10). As a result of PM filter application over RGB space, the red channel increases separation levels with respect to background, and this result is relevant as the application of PM filter improves stomata profile detection, as it is exemplified in Figure 11.

**Figure 12:**
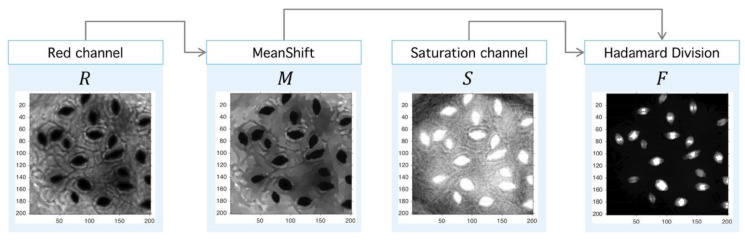
Image preprocessing with Meanshift-Hadamard division. After the PM usage (see Figure 10), the filtered red-channel $\partial_{t}P^{R}$ is used as input for Meanshift; the output $M\left(\partial_{t}P^{R}\right)$ is combined with the saturation channel of the original image $P^{S}$ through the Hadamard division, and this later image is subject to clustering.

**Step II.** Meanshift-Hadamard: The second step is the application of Meanshift M operator over the red channel to obtain an image with a reduced intensity level but with sharp defined boundaries, which eases the segmentation process. This step uses an unsupervised clustering algorithm over the intensity-space through a minimization process between each energy cluster [47]. With the aim at improving signal-background splitting, a Hadamard division [48] operator between saturation (HSV space) and red (RGB space) channels is applied. This operation enhances signal splitting, as it can be seen in Figure 12. The final operator is the consecutive application of PM, Meanshift and Hadamard division (3):

(3) $$F\;=P^{s}\;⊘M\left(\partial_{t}P^{R}\right),$$

where F is the resultant preprocessed image.

This two-step algorithm might be modified based upon stomata-image type. However, the proposed technique is useful as segmentation method as it helps with the post-labeling process, which is a main focus of the research.

### 4.2. Delaunay-Rayleigh Threshold Binarization (DRTB Algorithm)

**Figure 13:**
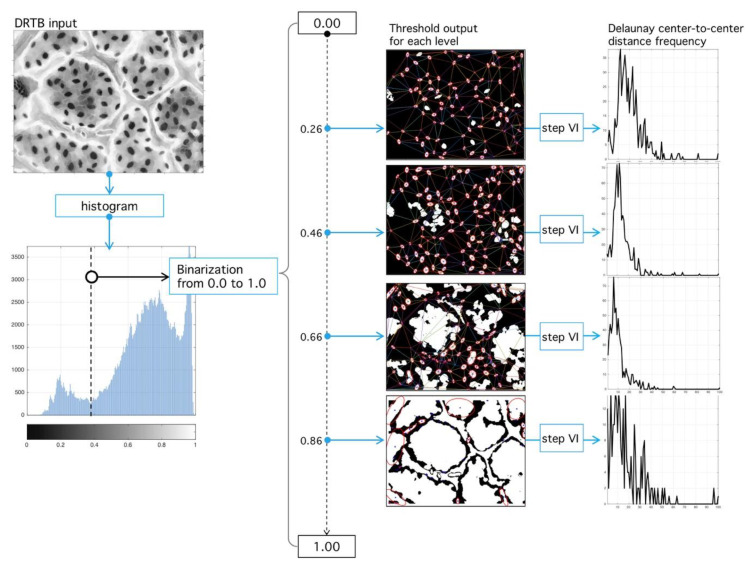
Binary segmentation and Delaunay tessellation. (Left) Grey-scale image (see Figure 12) is subject to the process of binarization at the l-level. (Right) Different binarization threshold results in different tessellations. The best tessellation is found through an optimization procedure applied over the RMSD, with respect to an ideal Rayleigh distribution and the empirical histogram.

Our main algorithm uses stomata spatial localization as a method to find an optimal binarization threshold. As input, it uses the grey-scale image F and outputs an optimal umbralization level such that error in Delaunay-distance frequencies be minimum with respect to an ideal Rayleigh distribution [28] (Figure 13). Next, we present detailed description of DRTB algorithm phases.

**Step III.** Threshold level binarization: First, phase is binary-image building given a threshold. Literature gives various binarization techniques; however, most of them use as input information the relationship between intensity levels of the image. Instead, our algorithm explores a geometric-analysis of image embedded structures as optimal binarization-level search method. Initially, all binarization level-space values are traversed by normalization of intensity-levels in [0,1] domain and then we explore such space. Binarization operator ${ℋ}_{l}\left(F\right)$ at level l is defined as:

(4) $${ℋ}_{l}\left(F\right)=\{\begin{array}{c} 0,\;\;F\leq l,\\ 1,\;\;F>l \end{array}$$

which has the same size of F but with values in [0,1]. All pixels with values higher that l are given the value 1 and the rest 0. From the image ${ℋ}_{l}\left(F\right)$, we can obtain a series of $n_{R}$ regions with areas given by $r=[r_{1},\;r_{2},\;r_{3},\;\ldots ,\;r_{n_{R}}]$.

**Step IV.** Binary labeling and filtering: Valid regions are those whose area be not an outlier. We propose [49] methodology for outlier detection. First, median absolute deviation (MAD) is determined as:

(5) MAD=b MEDIAN(r−MEDIAN(r)),

measured in pixels with value 1, and b=1.4826 is a normality constant. Area outlier detection criterion is defined as:

(6) $$\left|\frac{r_{i}-\;MEDIAN\left(r\right)\;}{MAD}\right|<3,\;i=1,\ldots n_{R}.$$

This means that all regions not fulfilling the criterion will be discarded in the following analysis.

**Step V.** Delaunay tessellation: This phase consists in the generation of a triangular tessellation for all regions defined as segmented inliers. Mass centers for each i-th region are determined through central moment estimation [50].

**Figure 14:**
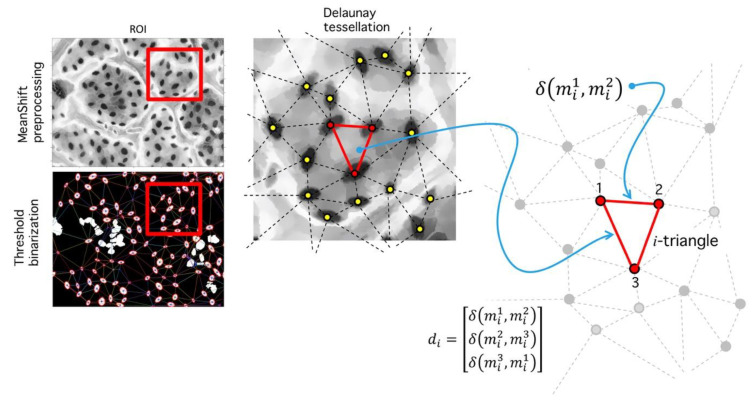
Delaunay tessellation over ROI (red square). (Left) Meanshift image and binarized image. (Center) Zoom over the ROI shows the segmented regions and its centroids (yellow dots). A Delaunay tessellation is built from centroids; note that centroid positions are dependent on the binarization level l. (Right) After the centroids are fixed and the tessellation is built, the set $d_{i}$ of all found distances are used to calculate a histogram, which is used for RMSD analysis.

Let $m_{i}$ the mass center corresponding to i-th region (see Figure 14). A Delaunay algorithm uses an iterative process to generate a triangular tessellation considering a given region. The output is a set of 3 vertices for each triangle in terms of its coordinates $\left[m_{i}^{1},m_{i}^{2},m_{i}^{3}\right]$; given a two-dimensional set of points, it is easy to build a tessellation joining the points (vertices) with lines (edges) in a triangulation. For each triangle, a circumcircle might be drawn. A Delaunay tessellation is such a triangulation that no vertex from the tessellation is found inside the circles. Figure 14 shows a triangulation composed by 17 vertices. For each triangle, its circumcircle is drawn. A circle meets three vertices, and no vertex is inside. Delaunay tessellations tend to maximize the smallest inner angle possible. Diffusion operators complies with a maximum principle, which are relevant in existence and uniqueness of solutions under this operation; it is possible to extend these properties to numerical solution calculations obtained over Delaunay tessellations, mainly because of the geometric regularity imposed over the angle distribution [51]. This kind of tessellation are used in partial differential equation analysis and morphogenesis studies, and we expect it might play a role given the importance of gas-diffusion in stomatal physiology.

**Step VI.** Distance analysis: As result of the last process, a set of triangles in obtained (Figure 14), each vertex an inlier-region. We analyze the set of all distances:

(7) $$d_{i}=\left[\delta \left(m_{i}^{1},m_{i}^{2}\right),\;\delta \left(m_{i}^{2},m_{i}^{3}\right),\delta \left(m_{i}^{1},m_{i}^{3}\right)\;\right],\;i=1,\;2,\;3,\ldots n,$$

where δ(·,·) is the Euclidian distance between vertices, and n is the number of tessellated triangles. Let $d=\left[d_{1},d_{2},\cdots ,d_{i},\;\cdots ,d_{3n}\right]$, a vector composed of all edges, and let $n_{i}$ the number of occurrences of distance $d_{i}$; then:

(8) $$p\left(d_{i}|l\right)=\frac{n_{i}}{\sum_{i=1}^{3n}n_{i}}$$

is the occurrence probability of $d_{i}$ in the vector d for a given threshold l obtained from the binarization process.

**Step VII.** Error estimation: Consider the Rayleigh distribution of parameter σ:

(9) $$ℛ\left(d_{i}|\sigma \right)=\frac{d_{i}}{\sigma^{2}}{ℯ}^{-\frac{d_{i}^{2}}{2\sigma^{2}}};$$

our experimental results point at its presence in all analyzed images (manual or automatic process); see Reference [28] for other applications of this probability in natural formations. Initial calibration points at σ=2 and t>0. By root mean-square (RMS) deviation (RMSD) minimization, we assess the proximity of $p\left(d_{i}|l\right)$ to $ℛ\left(d_{i}|\sigma \right)$ as a function of threshold l and σ-parameter:

(10) $$RMSD{\left(\sigma ,l\right)}_{}=\sqrt{\frac{\sum_{i=1}^{3n}{\left(ℛ\left(d_{i}|\sigma \right)-p\left(d_{i}|l\right)\right)}^{2}}{3n}}.$$

See the Discussion for comments on the significance of the parameter σ.

**Step VIII.** Optimal level selection: The ideal stomata distribution is associated with the minimum RMSD. The optimal parameter $\hat{\;l\;}$ happens when such distance is minimum:

(11) $$\hat{\;l\;}=\begin{array}{c} argmin\\ 0<l<100 \end{array}\left(RMSD_{\sigma ,l}\right).$$

**Figure 15:**
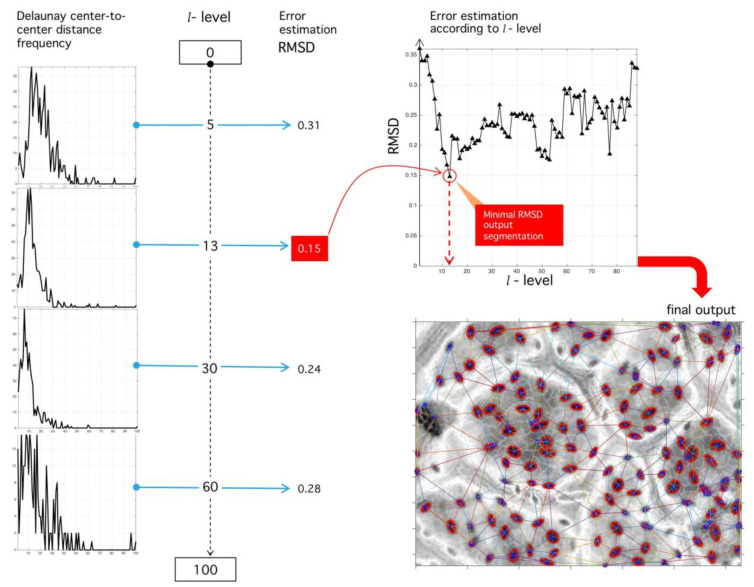
Sensibility analysis of binarization level and RMSD optimization procedure. (Left) Different histograms corresponding to different binarization levels. (Upper right) RMSD sensibility with respect to binarization level. The level of binarization minimizing the RMSD error is used for the final tessellation. (Lower right) Final output of the proposed algorithm with the positions of stomata fixed at tessellation nodes.

Once the best level is found, the image is finally binarized. An example is shown in Figure 15.

## 5. Conclusions

We showed an optimal segmentation algorithm based on stomata-position frequency-analysis through Delaunay tessellation and Rayleigh distribution. We tested the algorithm with *Hymenaea Courbaril* before a preprocess technique aimed at noise reduction. Our proposal is centered around a Delaunay-Rayleigh Threshold Binarization (DRTB algorithm) that allows an optimal binarization threshold with a posterior segmentation. The automatic segmentation shows the presence of a stable Rayleigh distribution, despite image differences. This result is relevant given that DRTB algorithm might be applied to other species, as was shown in the manual experimental phase with seven different species.

## Figures and Tables

**Table 1:** Stability of Rayleigh parameter for various species. From 2842 regions analyzed, a stability range 3<σ<8 might be proposed. Maximum Rayleigh parameter is 11.1 (*Tradescantia Pallida*) minimum value is 3.3 (*Tradescantia Zebrina*). Family name: C: *Commelinaceae*, M: *Marantaceae*, F: *Fabaceae*.

#	Species Analyzed	Stomata’s Number	RMSD	Rayleigh Parameter	Group	Family
1	*Tradescantia Zebrina*	753	0.00832	3.3	*Monocot*	C
2	*Tradescantia Pallida*—under 24 h of light	394	0.00983	3.6	*Monocot*	C
3	*Tradescantia Pallida*—in natural condition	245	0.03327	7.1	*Monocot*	C
4	*Callisia reppens*	65	0.05358	8.8	*Monocot*	C
5	*Callisia reppens*	29	0.12969	9.7	*Monocot*	C
6	*Callisia reppens*	104	0.02037	4.3	*Monocot*	C
7	*Tradescantia Zebrina*	69	0.05905	8.3	*Monocot*	C
8	*Tradescantia Pallid*	25	0.13832	11.1	*Monocot*	C
9	*Ctenanthe Oppenheimiana*	138	0.02531	7.1	*Monocot*	M
10	*Calisia reppens*—using stereoscope 15×	586	0.01578	3.3	*Monocot*	C
11	*Tradescantia Pallida* using stereoscope 15×	295	0.01902	5.4	*Monocot*	C
12	*Hymenaea Courbaril*	139	0.02420	6.1	*Dicot*	F
	Total Regions	2842	μ = 0.04473			

## Supplementary Materials

Our solution can be accessed online at https://github.com/mlacarrasco/drtb, and images database are available online at https://github.com/mlacarrasco/drtb/tree/main/database.
